# Innovative Approaches to Material Selection and Testing in Additive Manufacturing

**DOI:** 10.3390/ma18010144

**Published:** 2025-01-02

**Authors:** Alexandr Fales, Vít Černohlávek, Jan Štěrba, Milan Dian, Marcin Suszyński

**Affiliations:** 1 Faculty of Mechanical Engineering, Jan Evangelista Purkyne University in Ustí nad Labem, Pasteurova 1, 40096 Ustí nad Labem, Czech Republic; alexandr.fales@ujep.cz (A.F.); jan.sterba@ujep.cz (J.Š.); milan.dian@ujep.cz (M.D.); 2 Institute of Mechanical Technology, Poznan University of Technology, 60-965 Poznan, Poland; marcin.suszynski@put.poznan.pl

**Keywords:** 3D printing, filament, PLA, PETG, ASA, ABS, model, kits, robotics, VEX, VEX IQ, education, destructive tests, tensile load, deflection

## Abstract

This study focuses on selecting a suitable 3D printer and defining experimental methods to gather the necessary data for determining the optimal filament material for printing components of the VEX GO and VEX IQ robotic kits. The aim is to obtain the required data to identify an appropriate filament material and set 3D printing parameters to achieve the desired mechanical properties of the parts while maintaining cost-effectiveness. Another key objective is achieving optimal operational functionality, ensuring the required part performance with minimal printing costs. It is desirable for the modeled and printed parts to exhibit the required mechanical properties while maintaining economic efficiency. Another crucial aspect is achieving optimal functionality of the produced parts with minimal printing costs. This will be assessed by analyzing the impact of key 3D printing technology parameters, focusing in this research phase on material selection. The criteria for selecting filament materials include ease of printability under the conditions of primary and secondary schools, simplicity of printing, minimal need for post-processing, and adequate mechanical properties verified through experimental measurements and destructive tests on original parts from VEX GO and VEX IQ kits. The study analyzed various filaments regarding their mechanical properties, printability, and cost-effectiveness. The most significant practical contribution of this study is selecting a suitable filament material tested through a set of destructive tests, emphasizing maintaining the mechanical properties required for the real-life application of the parts. This includes repetitive assembly and disassembly of various robotic model constructions and their activation for demonstration purposes and applications of STEM/STEAM/STREAM methods in the educational process to achieve the properties of original components. Additionally, the study aims to set up 3D printing such that even a beginner-level operator, such as a primary or secondary school student under the supervision of their teacher or a teacher with minimal knowledge and experience in 3D printing, can successfully execute it. Further ongoing research focuses on evaluating the effects of characteristic 3D printing parameters, such as infill and perimeter, on the properties of 3D-printed parts through additional measurements and analyses.

## 1. Introduction

Current advances in 3D printing and additive technologies: Recent advancements in 3D printing and additive technologies provide a wide range of materials suitable for applications in industries such as manufacturing, construction, healthcare, education, and household use. Among the primary 3D printing technologies is Fused Deposition Modeling (FDM), which utilizes filaments as the printing material. Continuous development in filaments and printers aligns with the growing demands of 3D printing, enabling the production of objects with mechanical, thermal, and radiation resistance; large and complex structures; and interconnected, movable parts without the need for adhesives, welding, or fasteners, including the integration of magnets. The overall capabilities of 3D printing are thus driven by customer needs [1].

Filaments are currently the most widely used material for printing on FDM printers. These materials are polymers, specifically thermoplastics [2]. Polymers are macromolecular substances composed of chains of molecules consisting of repeating structural units called monomers, which are linked by covalent bonds to form macromolecules with high molecular weight. The chemical structure of polymers can be linear, branched, or crosslinked, influencing their physical and chemical properties. Polymers can be categorized as natural or synthetic. Natural polymers include cellulose, starch, and proteins, while synthetic polymers are produced through chemical processes and include materials like polyethylene, polypropylene, and polystyrene [2].

Key properties of polymers include flexibility, thermal and chemical resistance, low density, and easy formability. These characteristics make polymers highly useful across a wide range of applications, including the production of plastics, textiles, medical devices, and electronics [3,4,5].

Polymers can further be classified into plastics and elastomers. Elastomers can be subdivided into rubbers and thermoplastic elastomers, while plastics are divided into thermoplastics and thermosetting plastics. Among these, thermoplastics are the most important and widely used materials in additive FDM technology.

Thermoplastics such as polyethylene (PE), polypropylene (PP), polystyrene (PS), polyethylene terephthalate (PET), polyvinyl chloride (PVC), and polyamide (PA) are heat-softenable materials. When heated to their melting temperature, they soften and enter a plastic state, which allows them to be shaped by bending, pressing, drawing, or blow-molding. With further heating, thermoplastics transition into a liquid state, enabling processing via injection molding. These materials become solid and rigid upon cooling, with the process being entirely reversible without causing permanent changes to the chemical structure or technological properties of the material. This reversibility allows thermoplastics to be recycled and reused. However, with increased reuse, chain degradation occurs, and the repeatedly melted thermoplastic, referred to as a recycled material, exhibits inferior properties.

Thermoplastics consist of linear, branched, or spatial macromolecules with varying structures, which further categorize them as amorphous or semi-crystalline. Amorphous thermoplastics are characterized by irregularly arranged chains and random macromolecular positions. These materials are very strong, with a low refractive index, enabling transparency or clarity. Typical parameters include modulus of elasticity, hardness, brittleness, and low shrinkage. Semi-crystalline thermoplastics exhibit crystallinity, which is the degree of order in their structure. They are milky in appearance and offer an advantageous combination of strength and toughness, although they have higher shrinkage [3,4].

Polymers play a critical role in 3D printing, particularly in methods such as Fused Deposition Modeling (FDM) and Selective Laser Sintering (SLS). These materials, composed of long molecular chains, offer a wide range of properties that enable their use in diverse applications. Advantages of polymers in 3D printing: design flexibility, cost-effectiveness, and material variety.

Commonly used polymers in 3D printing:

PLA (Polylactic Acid): This biodegradable polymer is popular for its ease of printing, low melting temperature, and eco-friendliness. PLA is suitable for prototype manufacturing, educational purposes, and decorative objects.

ABS (Acrylonitrile Butadiene Styrene): Known for its strength, impact resistance, and thermal stability, ABS is widely used in industrial applications, toy manufacturing, and functional parts.

PETG (Polyethylene Terephthalate Glycol): PETG combines the ease of printing offered by PLA with the strength of ABS, making it a versatile material. It is resistant to moisture and chemicals, making it ideal for practical and functional products.

Nylon (Polyamide): Valued for its high strength, flexibility, and wear resistance, nylon is suitable for technical applications, such as mechanical components and tools.

TPU (Thermoplastic Polyurethane): This flexible and elastic polymer is used to produce parts like seals, springs, and protective covers.

3D printing can significantly contribute to sustainability through several key aspects. Firstly, it facilitates waste reduction, as it enables the creation of parts with minimal material waste by using only the exact amount of material needed. Secondly, it supports material recycling, with numerous 3D printers capable of printing using recycled materials, thereby reducing the demand for new raw materials and lowering the ecological footprint. An interesting aspect is local manufacturing, which reduces the need for transportation and, consequently, the associated greenhouse gas emissions. Additionally, prototyping and design optimization, including lightweight and energy-efficient product designs, allow for the quick identification and correction of errors, thereby enhancing sustainability throughout the product’s lifecycle. Finally, 3D printing ensures material and energy savings. Due to its efficient use of materials and energy-efficient processes, it is generally more resource-efficient compared to some conventional manufacturing methods, making it a clear contributor to sustainable production practices [6].

The integration of 3D printing into education brings numerous positive impacts on the learning process. One of the most significant is hands-on learning, as students have the opportunity to apply theoretical knowledge in practice, leading to better understanding and retention of information. Students become more motivated and engaged, increasing their interest in the subject matter. 3D printing also fosters the development of critical thinking and problem-solving skills, as students learn to design and address challenges, promoting their ability to tackle complex tasks. Another substantial benefit is the enhancement of creativity, enabling students to experiment and generate new ideas, thereby supporting their innovative and creative thinking.

The integration of 3D printing allows for the realization of interdisciplinary projects that connect various subjects. For example, in art and technology, students can design and create artistic works using 3D printing. Similarly, in robotics and engineering, students can produce components for robots and machines, fostering their interest in these fields. Students also develop technical skills that may prove valuable in their future careers. 3D printing provides opportunities for solving real-world problems, as students can work on projects addressing actual challenges, such as creating prototypes for environmental initiatives or medical devices.

Lastly, 3D printing supports the development of collaboration and teamwork, including advocating for and defending their own ideas. This prepares students for future challenges in both their personal and professional lives, equipping them with essential skills for success.

### 1.1. Overview of Types of Materials for 3D Printing

The following is an overview of the types of materials for 3D printing as presented by various manufacturers and distributors on their websites. The classification is designed to cover the full range of these materials and serves as a guide. Specifically, 3D printing materials can be categorized as follows [5], with the possibility of further expansion to include additional materials [1,2,3,4,5,6,7,8,9,10,11,12,13,14]:

(1) Filaments (printing strings):
  - PLA (Polylactic acid)
    - PLA+
    - PLA—D
    - STEEL PLA/PLA BRONZE/PLA COPPER
    - Magnetic Iron PLA
    - PLA/PHA
    - PolyTerra PLA
    - LW PLA
    - PLA HD
    - PLA Plaster
    - PLA Conductive
  - PET (Polyethylene terephtalate)
  - PETG (Polyethylene Terephthalate Glycol-Modified)
  - ABS (Acrylonitrile Butadiene Styrene)
    - ABS+ (Ice filaments)
    - ABSi (Methyl Methacrylate Acrylonitrile Butadiene Styrene Copolymer)
    - ABS—T (Acrylonitrile Butadiene Styrene—Methyl Methacrylate)
    - ABS—ESD7 (Electrostatic Dissipative ABS)
    - ABS—M30 (Acrylonitrile Butadiene Styrene)
    - ABS Medical—M30i (Biocompatible Acrylonitrile Butadiene Styrene)
    - ABS F. P.
  - ASA (Acrylonitrile Styrene Acrylate)
  - IGUS (Tribo-filament)
  - Rubber (Flexible)
    - TPE—O: Thermoplastic Polyolefins (TPO)
    - TPE—S: Styrene-Based Blends of Polyolefin and SBS, SEBS, or SEPS (TPS)
    - TPE—V: Vulcanized PP/EPDM Blend PP/EPDM (TPV)
    - TPE—E: Copolyester Blend (TPE)
    - TPE—U: Thermoplastic Polyurethane (TPU)
    - TPE—A: Thermoplastic Polyamide (TPA)
  - SILK (Silky Gloss)
  - CPE (Copolyester)
  - HIPS (High impact polystyrene)
  - PA (Polyamide)
  - PC (Polycarbonate)
  - PP (Polypropylene)
  - PVC (Vinyl 303)
  - PVA (Polyvinyl Alcohol)
  - PVB (Polyvinyl butyral)
  - POM (Polyoxymethylene)
  - PMMA (Polymethyl Methacrylate)
  - SBS (Polystyrene Butadiene Styrene)
  - BVOH (Butenediol Vinyl Alcohol Copolymer)
  - ULTEM/PEI (Polyetheremide)
    - ULTEM 1010
    - ULTEM 9085
  - PEEK (Polyether Ether Ketone)
  - PCTG (Polycyclohexylenedimethylene Terephthalate)
  - METAL
    - Brassfill (Brass Filament)
    - Bronzefill (Bronze Filament)
    - Copperfil (Copper Filament)
    - Steelfill (Steel Filament)
  - Glow in the Dark
  - Special
  - Composites
  - XT (Amorphous Copolymer)
  - Bamboofill (Bamboo-Filled Filament)
  - Woodfill (Wood-Filled Filament)
  - Eco (Recycled Materials)
(2) Resins
  - Standard
    - Hard
    - Model
    - BioBased
  - Medical
  - Engineering
    - Casting
    - Flexible
  - Cleaners
(3) SLS Powders
(4) Granules
  - Pigments
  - Pellets

### 1.2. Filaments

Filament, generally referred to as printing filament or the material used by a 3D printer for producing objects, prototypes, and products, is designed for 3D printing using the FDM method. Printing involves extrusion through a nozzle [5,8]. Filaments are polymer-based printing materials for FDM technology, suitable for both open-frame and enclosed 3D printers. The standard filament diameter is available in two sizes: 1.75 mm and 2.85 mm (commonly referred to as 3 mm) [10,12].

Filaments are typically sold as spools weighing between 200 g and 8 kg, though sample packs of standalone filament strands (about 100 g) can also be purchased [10,12]. Increasingly, manufacturers offer variants labeled as “Refill”. These are usually spools containing 800 g to 2 kg of coiled filament without an inner spool. This type of filament can be mounted onto an empty reusable spool with removable and reattachable side plates. This approach not only saves costs on spool purchases but also aligns with sustainability efforts to reduce the environmental impact of plastic waste.

Most filaments consist of pure plastic materials, but the market is increasingly offering plastic filaments with various additives or filaments made from non-plastic materials [8].

### 1.3. Essential Properties and Parameters

When selecting filament for 3D printing, the key factor is the object being printed. Additionally, the requirements for mechanical, thermal, and other properties must be considered. The choice of filament is also influenced by the 3D printer and its components, such as the nozzle and bed, on which the object will be printed. The reason for choosing the correct filament is simple: to avoid potential problems that could affect the print quality and the final appearance of the print, potentially requiring post-processing [15].

Filament diameter—The most common diameters are 1.75 mm and 2.85 (3) mm. It is essential to ensure compatibility with the printer.

Material of filament—This relates to the printed object, i.e., the required properties must align with the filament material.

Maximum nozzle temperature in °C—The melting point varies for different materials used in 3D printing. This determines print quality and potential printer damage. The nozzle temperature must be adequate for melting the filament and allowing proper extrusion without it solidifying prematurely. Lower nozzle temperatures result in slower melting, leading to better print quality but longer print times. Conversely, higher nozzle temperatures speed up melting but can lower print quality with shorter print times.

Print bed temperature in °C—This defines the optimal printing conditions. The heated bed helps to prevent shrinkage or deformation of some materials during the cooling process. The ideal temperature depends on the filament material used.

Flexibility—This property is related to the mechanical stress the object will undergo. For objects placed near a television or on a shelf, flexibility may not be crucial. However, for functional, stressed parts, higher flexibility is necessary to prevent breaking.

Thermal resistance—This property relates to the temperature range to which the printed object will be exposed. Higher thermal resistance means the object will maintain its shape under higher temperatures, whereas lower resistance means it will soften or melt. Consider where the object will be placed, especially in hot environments, to avoid degradation [16,17,18,19].

Odor—Some materials release unpleasant fumes during printing.

Toxicity of fumes—Caution is required as some materials release harmful fumes, which can cause nausea, vomiting, or fainting. Adequate ventilation is crucial during printing, and specialized enclosures with fume extraction should be used when necessary.

Environmental biodegradability—Biodegradable materials are environmentally friendly, making them ideal for support structures that are discarded after printing.

Recyclability—Materials that can be reused in the production process are considered eco-friendly, supporting sustainable 3D printing. These include eco-labeled materials made from recycled plastics.

Food and water contact—Most 3D printing materials are not suitable for long-term contact with food or drink, as they may contaminate the items with toxic substances or bacteria, potentially causing health issues.

Printability—Not all filaments are the same. A filament from one manufacturer may differ from the same material from another, as manufacturers use different production processes and additives. This can affect printability and may require adjusting the print profile. Some manufacturers provide recommended profiles for their filaments.

Additionally, the print bed (or platform) needs to be carefully managed. A detachable magnetic PrintPad is typically used. The first layer and material must adhere well to the print bed; otherwise, previously printed layers may detach, resulting in a failed print. It is crucial to ensure that no contaminants, especially oils from previous prints or handling, are present on the print bed. The bed can be cleaned with isopropyl alcohol (IPA) to ensure good adhesion.

Different filaments may require specific print bed surfaces or treatments to improve adhesion. For instance, for low-adhesion filaments like PLA, a smooth PEI-coated print bed is sufficient. To improve adhesion for flexible materials, adhesives such as 3DLAC or other products like Kores tube glue or double-sided tape may be necessary. High-adhesion materials, on the other hand, may require a satin or rougher print bed surface or the use of a separation layer.

After printing, the bed should be allowed to cool fully to easily remove the prints.

In a study to understand what filaments are used by primary and secondary schools for 3D printing, it was found that schools typically prioritize cost over quality. As a result, they often purchase different brands of filaments due to market surveys or tender requirements, leading to issues such as poor print quality, clogs, and nozzle or print bed damage. A key conclusion from discussions in professional forums and blogs is that filament quality should take precedence over price. Popular high-quality filaments in schools include Prusament, Fiber3D, and PM Filament. Additionally, it is recommended that different printers be dedicated to different filament types, such as PLA on one printer and PET-G on another, to avoid maintenance issues. Unfortunately, this practice is often overlooked in schools, leading to increased printer maintenance problems [20,21,22,23,24].

## 2. Selection and Description of Materials

The decisive parameters for selecting filament material are ease of printing, excellent mechanical properties of the print, minimal post-processing, and filament quality from a trusted manufacturer to ensure the print succeeds on the first attempt and to prevent issues such as nozzle clogs. Last but not least, due to the funding constraints of Czech education, price is also a determining factor. The manufacturers tracked were Prusament, Fiber3D, and Filament PM. From the materials available on the market that meet the observed criteria, the following filaments were selected: PLA (easy to print, weaker mechanical properties, no post-processing), PET-G (easy to print, better mechanical properties, minimal post-processing), ABS (more challenging to print, excellent mechanical properties, easy post-processing), and ASA (a replacement for ABS due to its lower toxicity and easier printing). The following is a detailed description of the selected materials for 3D printers with FDM technology.

The comparison of the FDM (Fused Deposition Modeling) 3D printing method with traditional manufacturing methods for plastic parts highlights several advantages. One of the primary benefits is design flexibility, as FDM allows the creation of complex and precise shapes that would be difficult or costly to produce using conventional methods. Additionally, FDM offers speed of production and cost efficiency for small series or individual parts, as there is no need to create molds, and only the exact amount of material required is used.

FDM is also ideal for prototyping, enabling the production of testable and modifiable models before final production. Furthermore, it supports the use of a wide range of materials, including various types of plastics and composites. On the other hand, there are some limitations. Certain materials may result in less smooth and detailed surfaces and exhibit lower mechanical properties and strength compared to parts produced by conventional methods. For large production series, conventionally manufactured parts become more cost-effective and faster to produce. A key drawback of FDM printing is the size limitation of parts, which is constrained by the build volume of the 3D printer. Lastly, there are highly specific materials that require printing conditions that cannot be achieved with FDM technology.

## 3. Measurements Performed

### 3.1. Printer Selection

The printability in the conditions of primary and secondary schools and the requirement for easy printing (beginner level) define printers using FDM (Fused Deposition Modeling) technology. During the research conducted to determine which robotic kits are currently used for educational robotics applications in primary and secondary schools in the Czech Republic, it was also investigated how 3D printing is implemented in these schools, which 3D printers are most commonly used in teaching, and which specific filaments they focus on and why. Additionally, during the research process, the level of knowledge and skills related to 3D printing was observed, and levels were defined for easier classification: novice, beginner, intermediate, advanced, and expert. The research was conducted through personal consultations during training sessions, webinars, and project days with school principals, vice-principals, and teachers who, at the time, were either using or preparing to implement 3D printing in their educational process. A total of 2156 participants took part. The research clearly shows that, currently, the most commonly used 3D printers in primary and secondary schools are from Prusa Research, a company founded in 2012 by Czech developer and maker Josef Průša. Today, over 700 people work there, and every month, more than 10,000 Original Prusa printers are shipped from Prague to 160 countries worldwide. The research revealed that these are primarily FDM technology printers, with a significantly smaller number of MSLA (masked SLA) printers found in secondary schools. When asked why Prusa printers are so widespread in Czech schools, the answer is simple. The reason is that Czech schools were once provided with Original Prusa i3 MK3S printers from the company as part of educational development support, practically free of charge, and today, they can receive, for example, the Original Prusa MINI+ printer completely free for educational purposes if they meet the required conditions set by the current call or program. A discount is also offered on selected devices if the buyer is a school or educational institution. Another reason for acquiring the mentioned printer is the well-developed methodological support from Prusa Research through a comprehensive portal that addresses all the challenges that a printer user might encounter during 3D printing. Last but not least, another significant reason is the unique support provided through a chat with experts, where any problem with 3D printing or a 3D printer can be solved 24/7, as professional printers from around the world are added to this section, responding immediately and offering knowledgeable advice on how to resolve the issue quickly. The quality of the filaments from this company is another strong argument. Some respondents also mentioned that it is a Czech company.

The research revealed that the most commonly used 3D printer platform in primary and secondary schools is from Prusa Research. The most frequently used printers from Prusa Research in Czech schools are as follows:

- Original Prusa MINI+
- Original Prusa i3 MK3S+
- Original Prusa MK4

**Figure 1:**
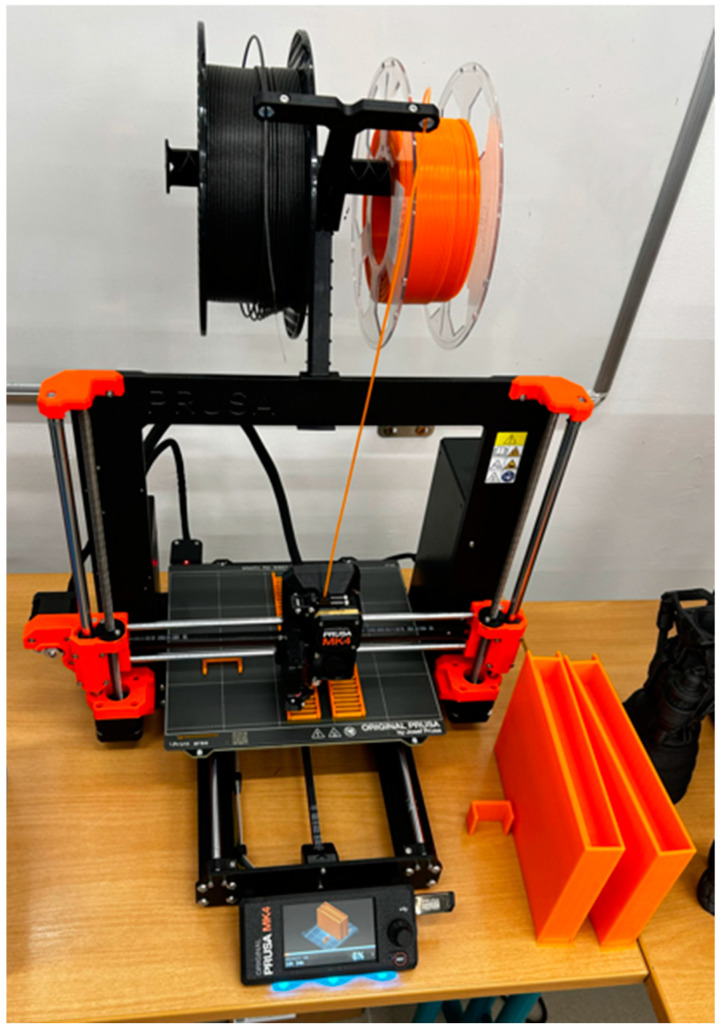
Printer Original Prusa MK4.

The last variant, the Original Prusa MK4, was chosen and is shown on Figure 1. The reason for this choice was that it is a newer version, and its evaluation is very positive compared to the other models. Last but not least, this printer is owned by our Faculty of Mechanical Engineering at our University, where the individual prints were carried out.

### 3.2. Selection and Modification of Parts for 3D Printing

As part of research focused on educational robotics and its utilization in primary and secondary schools, it was found that the most commonly used robotic kit platform at these schools is currently VEX, followed historically by LEGO. The VEX platform is predominantly utilized in its IQ 1st and 2nd generation versions, as well as the GO version. LEGO platforms commonly in use include WeDo 2.0, MINDSTORMS EV2 and EV3, and SPIKE.

Furthermore, in collaboration with AV Media Systems, a.s., and based on feedback from surveyed educators, the need emerged to enable the 3D printing of selected parts from the VEX IQ and GO robotic kits. Specifically, the goal was to develop a methodology that would allow teachers and their students at primary schools to 3D print selected VEX IQ and GO components with appropriate quality using the 3D printers and related resources available in Czech primary and secondary schools.

The selection of the printer and materials was described in the earlier section of the article. At this stage, it is necessary to determine the components suitable for 3D printing and subsequent destructive testing to ensure that the outcome is practical and meets the defined criteria.

The reasons for choosing the VEX GO and VEX IQ kits can be found in the article [12]. By comparing the two types of kits, the VEX GO kit with 275 pieces and the VEX IQ kit with 1000 pieces, two suitable parts were selected. The criteria for choosing the appropriate part were as follows:

- It is found in both types of kits, both VEX GO and VEX IQ.
- It has suitable dimensions for mounting in machines and fixtures for experimental measurements and testing in terms of destructive tests involving tensile load and deflection.

**Figure 2:**
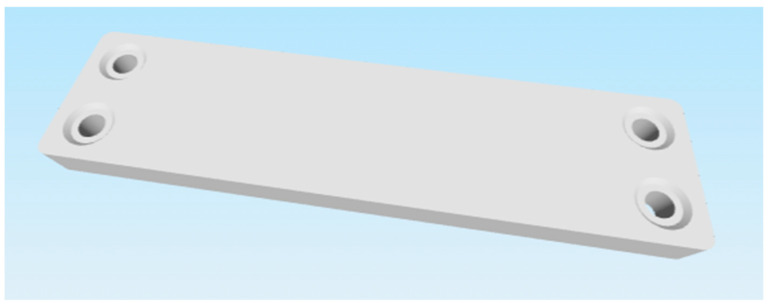
Original part 2 × 8 Smooth Panel (228-2500-524) VEX—top side.

**Figure 3:**
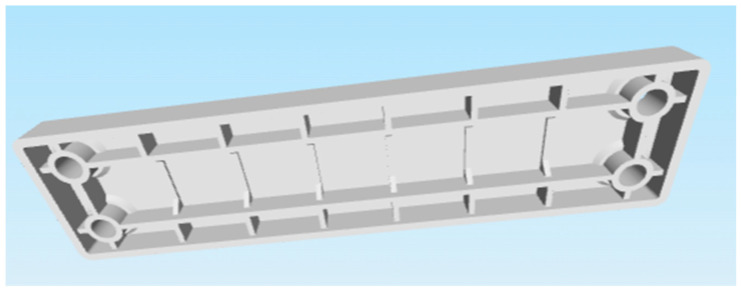
Original part 2 × 8 Smooth Panel (228-2500-524) VEX—bottom side.

**Figure 4:**
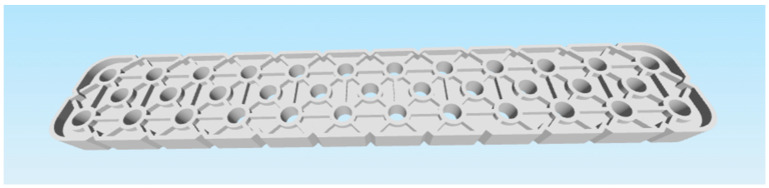
Original part 2 × 12 Beam (228-2500-026) VEX—top side.

**Figure 5:**
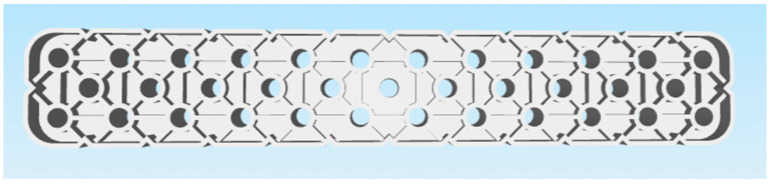
Original part 2 × 12 Beam (228-2500-026) VEX—bottom side.

These criteria led to the selection of the following two parts:

- Selected part: 2 × 8 Smooth Panel (228-2500-524) shown on Figure 2 and Figure 3.
- Selected part: 2 × 12 Beam (228-2500-026) shown on Figure 4 and Figure 5.

The selected parts, 2 × 8 Smooth Panel (228-2500-524) and 2 × 12 Beam (228-2500-026), were subsequently prepared for attachment to the machine and fixture for experimental measurements and testing, specifically for destructive testing under tensile load and deflection. The 2 × 8 Smooth Panel (228-2500-524) was found to be completely unsuitable for this type of experimental measurement and testing. On the other hand, the 2 × 12 Beam (228-2500-026) fully met the requirements both dimensionally and in terms of attachment. For this reason, the 2 × 12 Beam (228-2500-026) was chosen as the final part for the intended 3D printing and subsequent experimental measurements. After the final selection, the parts were secured in the required quantities from the original VEX GO and IQ kits.

Special attention was given to the modification of the lower part of the model, which facilitated printing and eliminated the need for post-processing.

### 3.3. Modification of the Part Model for 3D Printing Purposes

**Figure 6:**
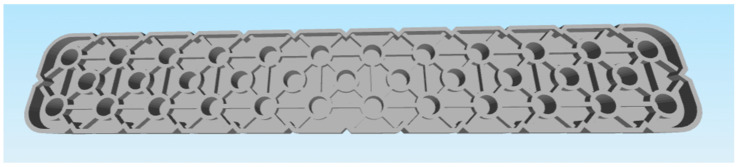
Modified part 2 × 12 Beam (228-2500-026) VEX—top side.

**Figure 7:**
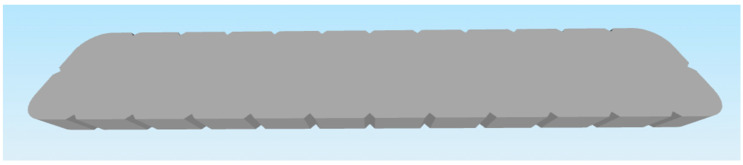
Modified part 2 × 12 Beam (228-2500-026) VEX—bottom side.

After the test print of the 2 × 12 Beam (228-2500-026) part, it was found that the model was unnecessarily complicated for subsequent postprocessing. Specifically, during slicing, an extensive support structure was created, which resulted in “unnecessary” material consumption, given the purpose of the part being printed for destructive experimental measurement and testing. Additionally, there was a significant time burden in removing these supports from the printed part. For this reason, it was necessary to modify the part’s model to ensure that the printing process was as quick and simple as possible, with minimal or no postprocessing required. The original model was adjusted by flattening the underside and blocking the holes for the pins. Shown on Figure 6 and Figure 7. In terms of testing, this adjustment does not have a significant impact on the results, as the dimensions and shape of the parts, printed from different filament materials, are identical.

## 4. Evaluation of Measurements and Discussion

### 4.1. Preparation of Prints

All four selected filament materials (PLA, PET-G, ABS, and ASA) will undergo experimental measurement and testing through destructive tests for tensile load and deflection. In terms of printing, the print will be made with 100% infill. The slicing will be performed using PrusaSlicer 2.7.4, with the printer settings selecting a nozzle-to-bed distance of 0.10 to 0.20 mm for higher detail, considering the model’s geometric complexity. This will also slow down the print speed, which is desirable. For printing speed, particular focus will be placed on the first layer, with the print speed reduced to 10 mm/s based on recommendations from professional 3D printers. The first layer is crucial for the entire printing process. Any imperfections or contamination of the surface, nozzle, or loose belts can significantly increase the risk of print failure, potentially leading to detachment of the print. In terms of pattern and fill size, a 100% linear infill will be used for the first print. These values are chosen with the consideration that these parts will be printed by students, teachers, and pupils at elementary and secondary schools, with varying levels of experience in 3D printing and varying equipment capabilities.

### 4.2. Tensile Load Test

**Figure 8:**
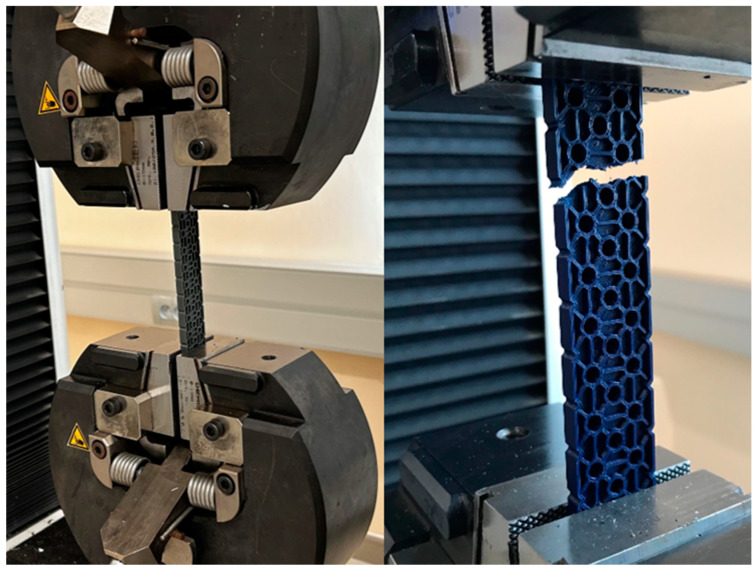
Static tensile test—before and after the test completion.

The static tensile test is a fundamental mechanical test used to determine the material’s mechanical properties, particularly its strength, elasticity, and plasticity. During this test, the material sample is subjected to unidirectional tension until failure. In the case of tensile loading, we determine the maximum force required to destroy the sample. The samples, including the original parts of the VEX IQ kit and 3D prints made from PLA, PET-G, ABS, and ASA, are secured in self-locking grips designed for flat samples (see Figure 8). Before starting the test, the force sensor and position are zeroed. The loading rate was set to 10 mm/min.

Two sets of samples were tested on two consecutive days, and two sets of results were recorded. The aim of the testing is to compare and evaluate the mechanical properties of the original VEX IQ part and parts printed from the following materials: 1 × PLA (Filament-PM PLA MarbleJet Light Marble 0.5 kg), 1 × PET-G1 (Fiber3D PETG Beige 1 kg), 1 × PET-G2 (Prusament PETG Jungle Green 1 kg), 1 × ASA (Prusament ASA Sapphire Blue 850 g), and 1 × ABS (SunLu ABS Filament Yellow 1 kg) on the Original Prusa MK4 3D printer. The experimental test will be conducted using tensile testing. The expected fracture location of the test prints is the part with two holes, or its immediate vicinity. The goal is to identify the material that performs best in the test, which will then undergo further experimental measurements and tests depending on the specific 3D printing settings.

**Figure 9:**
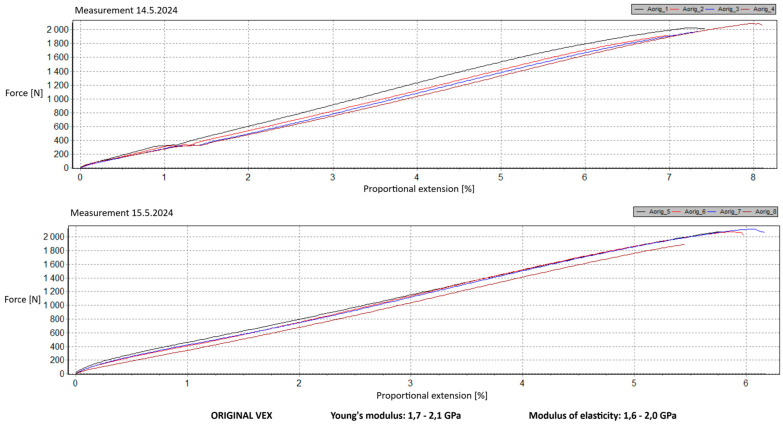
Tensile load test—multiple representations of the loading force curves—Original.

**Figure 10:**
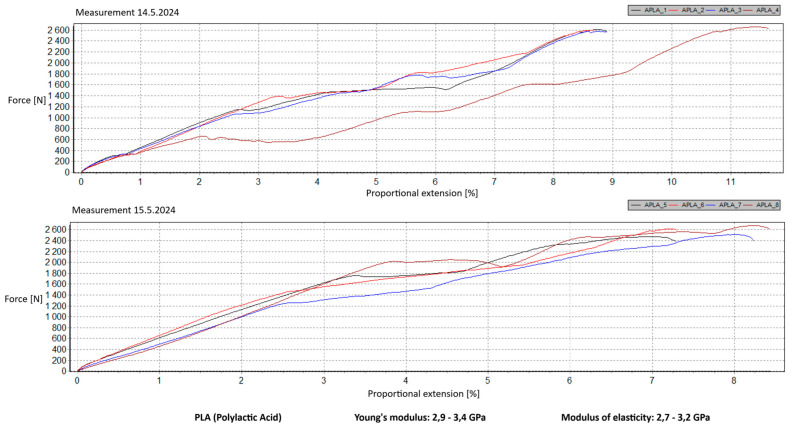
Tensile load test—multiple representations of the loading force curves—PLA.

**Figure 11:**
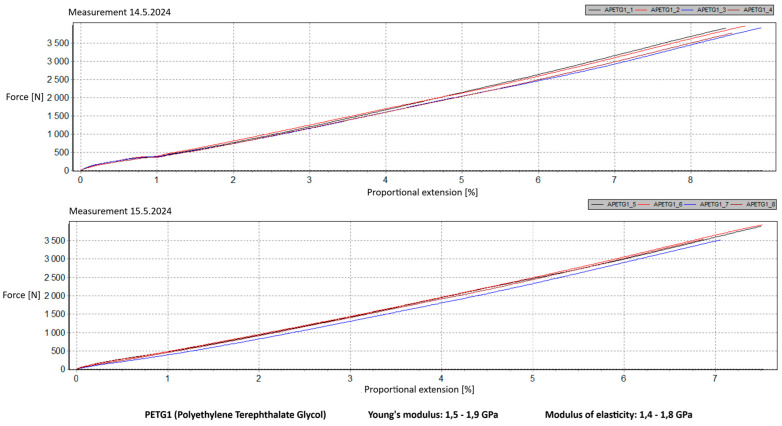
Tensile load test—multiple representations of the loading force curves—PET-G1.

**Figure 12:**
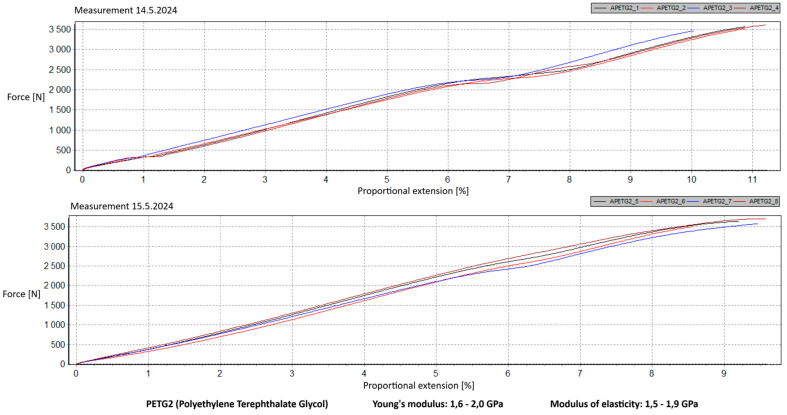
Tensile load test—multiple representations of the loading force curves—PET-G2.

**Figure 13:**
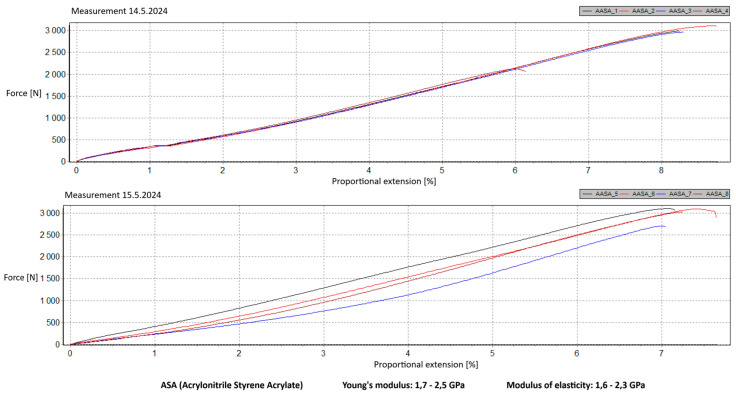
Tensile load test—multiple representations of the loading force curves—ASA.

**Figure 14:**
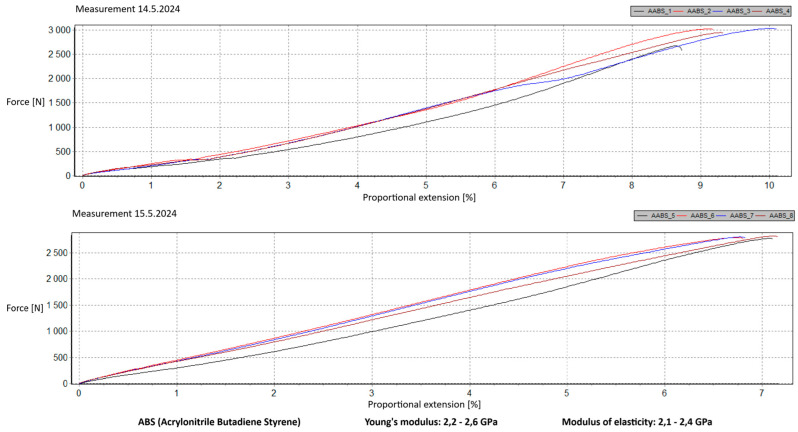
Tensile load test—multiple representations of the loading force curves—ABS.

Measurement Description and Procedure:

- Preparation of Test Prints—Visual inspection, removal of any stringing from PET-G, and labeling of test prints according to the table below.
- Setting Up the Tensile Testing Machine and Software Parameters (machine selection—Hegewald & Peschke inspect 100, selection of suitable clamping fixtures, pulling speed 10 mm/min, initial load F_0_ 2 N).
- Clamping the Test Print.
- Performing the Test—A total of two test sets were performed, each consisting of four samples, due to the time-consuming nature of the test and the workload of the destructive material testing laboratory at FSI, UJEP. In total, 8 tests were conducted for each material group. Since the resulting force did not vary significantly and the materials exhibited similar behavior, the test was concluded with 8 results, and no further tests were performed (see results in Table 1, Table 2, Table 3, Table 4, Table 5, Table 6, Table 7 and Table 8 and Figure 9, Figure 10, Figure 11, Figure 12, Figure 13 and Figure 14).
- Results Analysis—Fracture locations, maximum applied force at failure of the prints.

### 4.3. Deflection Test

**Figure 15:**
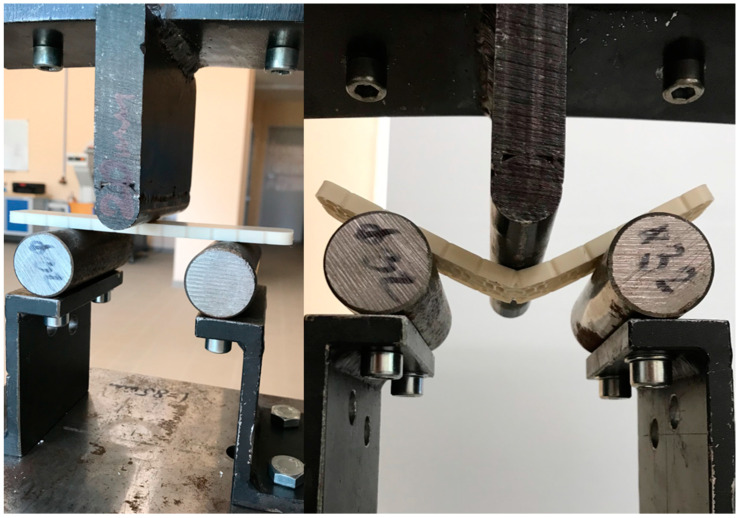
Deflection test—before and after the test.

As part of the testing, the samples are subjected to deflection loading. During the test, the samples, including original parts from the VEX IQ kit and 3D prints made from PLA, PET-G, ABS, and ASA, are supported by supports in the form of two rollers with a defined diameter. The entire sample is centered so that the load pin pushes at its center (see Figure 15). The test is concluded when a decrease in force is recorded on the graph. The results show the force required to reach the maximum deflection of the sample. Two sets of samples were tested on two consecutive days, and two sets of results were.

The goal of the testing is to compare and evaluate the mechanical properties of the original VEX IQ part and parts printed from the following materials: 1 × PLA (Filament-PM PLA MarbleJet light marble 0.5 kg), 1 × PET-G1 (Fiber3D PETG Beige 1 kg), 1 × PET-G2 (Prusament PETG Jungle Green 1 kg), 1 × ASA (Prusament ASA Sapphire Blue 850 g), and 1 × ABS (SunLu ABS Filament Yellow 1 kg) using the Original Prusa MK4 3D printer. The experimental test will be conducted using a deflection test. The expectation is that deflection will occur until the breakage of the individual printed parts at the point of pressure from the fixture. The material that performs best in the test will be selected for further experimental measurements and tests based on specific 3D printing settings.

Description and procedure of measurement

**Figure 16:**
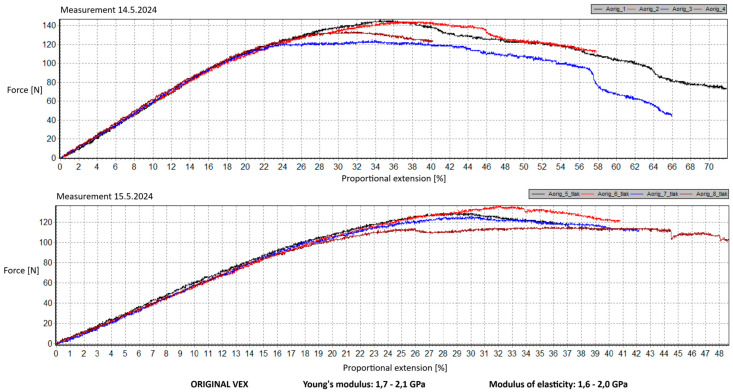
Deflection test—multiple representations of the applied force curves—Original.

**Figure 17:**
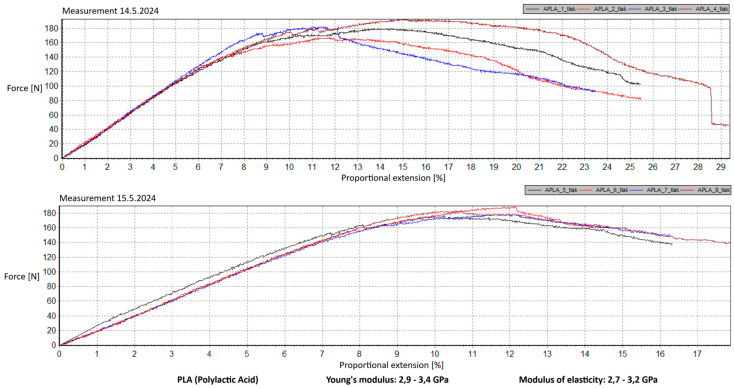
Deflection test—multiple representations of the applied force curves—PLA.

**Figure 18:**
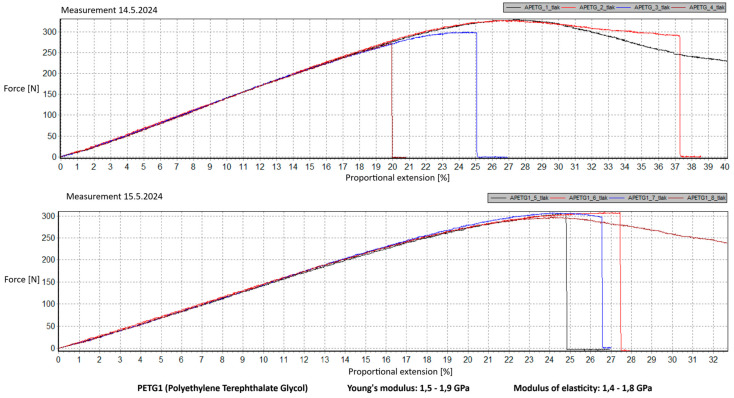
Deflection test—multiple representations of the applied force curves—PET-G1.

**Figure 19:**
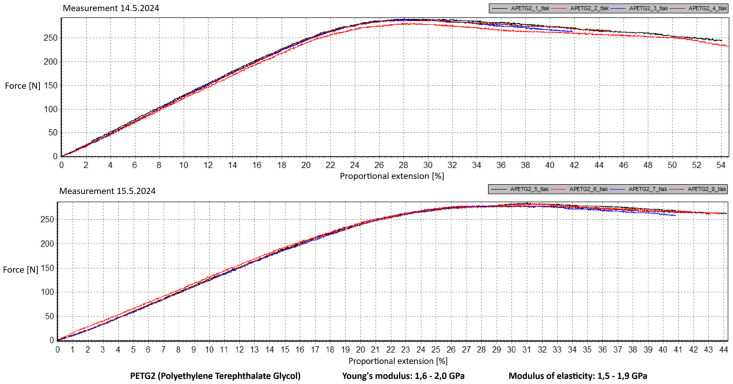
Deflection test—multiple representations of the applied force curves—PET-G2.

**Figure 20:**
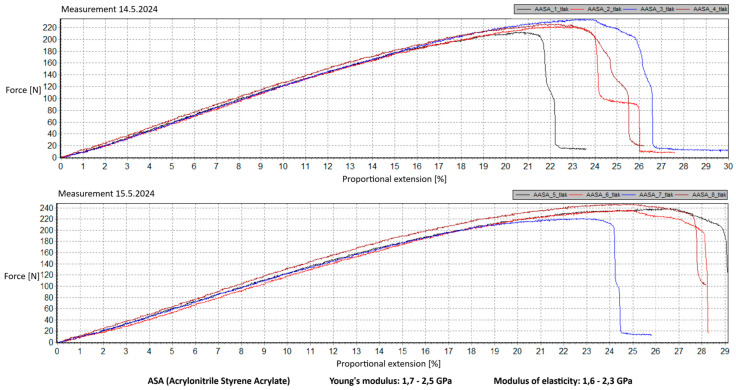
Deflection test—multiple representations of the applied force curves—ASA.

**Figure 21:**
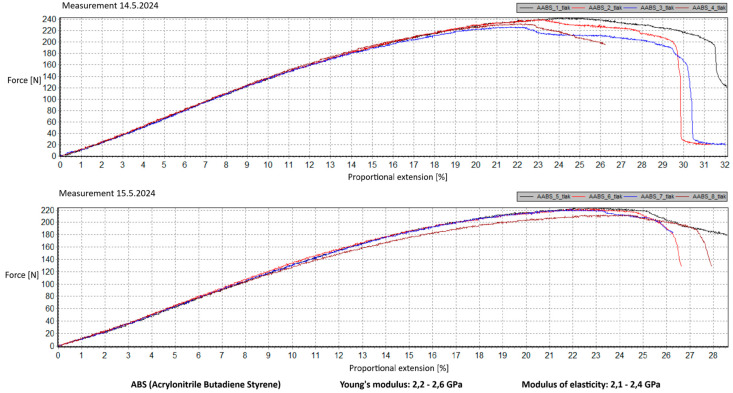
Deflection test—multiple representations of the applied force curves—ABS.

- Preparation of test prints—visual inspection, removal of any stringing on PET-G, labeling of test prints as per the table below.
- Setting up the testing machine and software parameters (machine selection—Hegewald & Peschke inspect 100, choosing appropriate clamping fixtures, pushing speed 10 mm/min, starting load F0 2 N).
- Clamping the test print.
- Conducting the test—two sets of tests were performed, each with 4 samples, considering the time and workload of the destructive materials testing laboratory at FSI, UJEP. A total of 8 tests were performed for each material group. Since the resulting force did not change significantly and the materials showed similar behavior, the test was concluded with 8 results, and no further testing was performer (see Table 9, Table 10, Table 11, Table 12, Table 13, Table 14, Table 15 and Table 16 and Figure 16, Figure 17, Figure 18, Figure 19, Figure 20 and Figure 21).
- Analysis of results—location of breaks, maximum loading force to maximum deflection or failure.

## 5. Conclusions

This section of the study focuses on analyzing the measured results of mechanical tests and subsequently selecting the optimal material for 3D printing selected parts of the VEX robotic kit, suitable for use in primary and secondary schools. Key evaluation factors included the maximum load force values and their comparison with the values of original parts. As part of the research, samples were printed from various materials using 3D printers, with the aim of determining how different materials affect the mechanical properties of the printed components.

As part of the selection of a suitable part from the original VEX GO and IQ robotic kit components, the 2 × 12 Beam (228-2500-026) was chosen, which fully meets the dimensional and mounting requirements for use in the Hegewald & Peschke Inspect 100 machine with clamping fixtures for performing destructive tensile load and deflection tests. The choice of these tests corresponds to the stress application principle for this part in the context of using the robotic kit in teaching at primary and secondary schools. The parts are primarily used for construction exercises when building selected robot models and subsequent disassembly, which aligns with their use in tensile load and deflection testing. The 2 × 12 Beam (228-2500-026) was slightly modified due to unnecessary complexity in post-processing. The modification involved aligning the bottom side and sealing the pinholes, reducing the time load for removing supports generated during slicing, and significantly lowering filament material consumption. This modification does not significantly affect the results of the testing, as dimensionally and shape-wise identical parts printed from different filaments are compared.

The choice of the 3D printer for printing parts of the robotic kit in primary and secondary school settings, with an emphasis on ease of printing (beginner level), led to the selection of a printer using FDM (Fused Deposition Modeling) technology by Prusa Research. Specifically, the Original Prusa MK4 printer was chosen. The reason for this choice is that Prusa Research printers are widely used in Czech primary and secondary schools, and the MK4 is a newer version with highly positive reviews compared to the other models monitored, such as the Original Prusa MINI+ and Original Prusa i3 MK3S+.

The selection of filament material was determined by key parameters: ease of printing, excellent mechanical properties of the print, minimal post-processing, quality of filament from a verified manufacturer, and cost. The selected filament brands were Prusament PLA, PET-G, ABS, and ASA.

For the experimental measurements and testing with destructive tests, all the mentioned materials were ultimately chosen. After conducting the necessary tests and destructive evaluations, the following assessments can be concluded.

Standardized tests for tensile load and deflection were selected for several reasons, including the availability of equipment, the relevance of results, and comparability with other research. The primary factor was equipment availability, as the Faculty of Mechanical Engineering possesses apparatus for standard mechanical testing, such as the Hegewald & Peschke Inspect 100, which, with appropriate fixtures, enabled deflection testing as well. This choice minimized the need for new investments or reliance on external laboratories, which would have significantly increased research costs—an approach neither the faculty nor the partnering company supported. The relevance of results was another key factor, as standardized tests for tensile load and deflection provide essential data on the mechanical properties of materials, directly applicable to evaluating the suitability of materials for the intended purpose. Specifically, these tests align with the goal of replicating the performance of original components under typical stresses encountered during student design exercises. Additionally, the use of standardized tests ensures comparability with other studies, facilitating the interpretation of results and ensuring alignment with widely accepted norms and methodologies. While alternative, specialized tests—such as dynamic mechanical analysis (DMA) or fatigue testing—could yield additional insights, they require specific equipment not currently available at the faculty and are limited by the constrained research budget. The selection of standardized tests was thus driven by practical and methodological considerations, ensuring the accessibility of equipment as well as the relevance and comparability of the findings.

### 5.1. Evaluation of the Tensile Load Test

This section evaluates the measured results and subsequently selects the appropriate material for 3D printing applications in primary and secondary schools. The measured data indicate that the maximum load force values for all 3D-printed parts exceed those of the original VEX components. This is due to the chosen infill pattern and the closure of the lower part of the model, which facilitates printing and eliminates the need for post-processing that could affect the final values of the mechanical test parameters.

Among the materials tested, PET-G prints from both manufacturers achieved the highest rankings. Specifically, PET-G2 material by Prusa Research, marketed as Prusament PETG Jungle Green 1 kg, demonstrated a smoother load force progression, a closer resemblance to the characteristics of the original VEX part, and superior printability. These attributes make it the ideal choice for use in primary and secondary school environments. Furthermore, PET-G2 material by Prusa Research shows the closest Young’s modulus and elasticity modulus values to the original VEX component, reinforcing its suitability. Due to its lower stiffness, this material provides the required flexibility and impact resistance, making it particularly well-suited for printing robotic kit parts that are subjected to repeated mechanical stress and require greater durability and elasticity.

In contrast, the higher stiffness values observed in PLA make it more appropriate for parts that do not require flexibility but prioritize precision and detail—criteria that are undesirable for the intended application. Additionally, during material testing, all PLA prints exhibited layer separation, a disadvantage for this specific use case.

Materials such as ABS and ASA demonstrated intermediate properties, consistent with manufacturer specifications. However, due to their challenging printability, the need for specialized equipment, stringent printing conditions, and at least intermediate-level skills from the operator, these materials are ultimately unsuitable for producing robotic kit components in primary and secondary school settings.

Lastly, the second PET-G material tested, Fiber3D PETG Beige 1 kg, exhibited significant fluctuations and abrupt breaks in stress–strain characteristics. Most printed parts experienced catastrophic failure, shattering into numerous small fragments under stress, making it entirely unsuitable for the intended application.

Although the tensile strength values were measured on prints made from filament with a 1.75 mm diameter, it is reasonable to assume that the mechanical properties of PET-G align with the requirements for 3D printing robotic kit components under the conditions present in primary and secondary schools. It is essential to emphasize that the test prints did not conform to any standardized shape. Instead, the measurements are specific to the geometry of the tested prints, which were designed to resemble the original VEX part as closely as possible. The intent was to ensure that these parts could be printed by students or teachers with minimal or no prior experience in 3D printing while maintaining mechanical properties similar to the original component.

### 5.2. Evaluation of the Deflection Test

This section of the study focuses on the analysis of measured results and the subsequent selection of a suitable material for 3D printing applications in primary and secondary schools. The results reveal that the maximum load force values for all printed samples exceed those of the original components. This outcome is attributed to the chosen infill structure and modifications to the lower part of the model, which were implemented to simplify the printing process and eliminate the need for post-processing, a factor that could potentially influence the mechanical test outcomes.

In terms of load force performance, PET-G materials from both manufacturers emerged as the most appropriate choice. Notably, the PET-G2 material by Prusa Research, specifically Prusament PETG Jungle Green 1 kg, demonstrated a more consistent load force progression and characteristics closely resembling the original VEX IQ part. Owing to its print quality, this material stands out as an optimal option. Although the deflection values were measured on prints made from 1.75 mm filament, it can be reasonably concluded that the mechanical properties of PET-G meet the requirements for producing robotic kit components in the context of primary and secondary education. Furthermore, PET-G2 material from Prusa Research exhibits Young’s modulus and elasticity modulus values closest to those of the original VEX part, further affirming its suitability. The lower modulus values ensure that the printed parts possess the necessary properties for this application, including enhanced flexibility and impact resistance. As a result, this material is highly suitable for manufacturing robotic kit components that must endure repeated mechanical stress.

Conversely, the higher modulus values observed in PLA make it better suited for parts that prioritize precision and detail over flexibility. However, these characteristics are undesirable for the intended application. Moreover, all PLA prints experienced layer delamination during mechanical testing, which is a significant drawback for this use case.

ABS and ASA materials exhibit intermediate modulus values, consistent with manufacturer specifications. However, their challenging printability, the need for specialized equipment, and stringent printing conditions render them unsuitable for producing robotic kit components in primary and secondary school settings.

The second PET-G material tested, Fiber3D PETG Beige 1 kg, displayed significant fluctuations and abrupt fractures in its stress–strain behavior. Most printed parts fractured catastrophically into numerous small fragments under stress, making this material entirely inappropriate for the intended application.

Although the tensile strength values were obtained from prints made using 1.75 mm filament, it can be inferred that the mechanical properties of PET-G align with the requirements for robotic kit production in primary and secondary schools. It is crucial to note that the test prints did not conform to any standardized geometry. Instead, the results are specific to the geometry of the tested parts, which were designed to closely replicate the original component. The aim was to ensure that these parts could be printed by students or teachers with minimal or no prior experience in 3D printing while maintaining mechanical properties similar to the original design.

### 5.3. Summary

Tensile Load and Deflection: The measured values demonstrated that the maximum load forces of all printed components exceeded those of the original parts. This phenomenon is primarily attributed to the use of an appropriate infill pattern, which provides additional support, and the optimization of the model’s lower section for easier printing. The analysis of results indicates that PET-G materials from both tested manufacturers achieved the best outcomes in terms of mechanical properties. In particular, the PET-G2 material from Prusa Research (Prusament PETG Jungle Green 1 kg) exhibited a smoother progression of load force and greater similarity to the original VEX part.

Prusament PETG Jungle Green emerged as the ideal choice due to its quality and stable mechanical properties. Although deflection values were measured on prints using a 1.75 mm filament, it can be assumed that the mechanical properties of this material meet the requirements for manufacturing robotic kit components in school environments.

The results of this testing confirm that the selection of the right material and infill type can significantly influence the mechanical properties of printed parts. PET-G, specifically Prusament PETG Jungle Green, appears to be the optimal choice for school environments due to its high strength, ease of printing, and minimal post-processing requirements. This material is well-suited for 3D printing robotic kit components in educational settings, offering optimal mechanical performance and ease of handling, which are essential for use in primary and secondary schools.

Finally, it is recommended to conduct further tests focusing on reducing printing costs in terms of filament consumption. The parameters to be monitored during printing will include the selection of the appropriate infill pattern and its density, as well as the influence of perimeters. Subsequently, additional materials from the current filament offerings will be tested.

This article serves as a pilot study in the selection and testing of materials such as PLA, PETG, ASA, and ABS. Future articles will build on this foundation, reflecting ongoing research focused on the potential applications of additional materials, including PC Blend, PCCF, PETG Tungsten 75%, Carbon Fiber Filament, Glass Fiber Filament, Wood Filament, and rPLA, with a comparison of their mechanical properties measured through tensile load and deflection tests. These tests are tailored to the specific types of stresses that robotic kit components are subjected to during their use.

In addition to this, further research will examine the impact of infill on the properties of 3D-printed parts, specifically investigating both the infill pattern and percentage density. These factors influence the internal support provided for the upper layers, which would otherwise need to bridge empty spaces. The overarching goal of this research is to reduce printing costs by minimizing filament consumption while maintaining the desired mechanical properties.

Infill is a critical element in 3D printing that directly affects the mechanical characteristics of printed parts. The ongoing research focuses on analyzing how various infill types and densities influence strength, toughness, and material usage. Patterns with the most significant impact on mechanical properties—such as grid, gyroid, and honeycomb—have been selected, with densities ranging from 10% to 100%. The objective is to identify the combination that most closely replicates the mechanical performance of the original VEX robotic kit component.

Another consideration is the minimization of filament consumption to reduce the overall cost of printing individual parts. This approach combines material efficiency with a focus on achieving optimal mechanical properties, aligning the research objectives with practical applications in educational and industrial settings.

## Figures and Tables

**Table 1:** Measured values from the tensile load test for Original item, PLA and PET-G1 materials.

Original VEX Part		Material PLA		Material PET-G1	
Print Designation	Loading Force F_max_ [N]	Print Designation	Loading Force F_max_ [N]	Print Designation	Loading Force F_max_ [N]
orig_1	2028	PLA_1	2611	PETG1_1	3906
orig_2	1927	PLA_2	2601	PETG1_2	3968
orig_3	1977	PLA_3	2579	PETG1_3	3917
orig_4	2090	PLA_4	2658	PETG1_4	3774
orig_5	2074	PLA_5	2474	PETG1_5	3895
orig_6	2077	PLA_6	2615	PETG1_6	3938
orig_7	2114	PLA_7	2508	PETG1_7	3523
orig_8	1889	PLA_8	2676	PETG1_8	3541
Arithmetic mean	2022	Arithmetic mean	2590.25	Arithmetic mean	3807.75

**Table 2:** Measured values from the tensile load test for PET-G2, ASA and ABS materials.

Material PET-G2		Material ASA		Material ABS	
Print Designation	Loading Force F_max_ [N]	Print Designation	Loading Force F_max_ [N]	Print Designation	Loading Force F_max_ [N]
PETG2_1	3568	ASA_1	2986	ABS_1	2674
PETG2_2	3513	ASA_2	3110	ABS_2	3020
PETG2_3	3458	ASA_3	2963	ABS_3	3032
PETG2_4	3609	ASA_4	2129	ABS_4	2946
PETG2_5	3638	ASA_5	3103	ABS_5	2775
PETG2_6	3506	ASA_6	3092	ABS_6	2791
PETG2_7	3579	ASA_7	2705	ABS_7	2804
PETG2_8	3700	ASA_8	3025	ABS_8	2820
Arithmetic mean	3571.375	Arithmetic mean	2889.125	Arithmetic mean	2857.75

**Table 3:** Calculation of the standard deviation of the loading force of the original part from the tensile load test.

Original VEX Part			
Print Designation	Loading Force F_max_ [N]	Δ F_i_	(Δ F_i_)^2^
orig_1	2028	6	36
orig_2	1927	−95	9025
orig_3	1977	−45	2025
orig_4	2090	68	4624
orig_5	2074	52	2704
orig_6	2077	55	3025
orig_7	2114	92	8464
orig_8	1889	−133	17,689
Arithmetic mean	2022	0	47,592
The standard deviation of the maximum loading force F_max_ for the printout			82.46
The resulting value of the maximum loading force F_max_ and its standard deviation			2022 ± 82.46 N

**Table 4:** Calculation of the standard deviation of the loading force for the PLA material from the tensile load test.

Material PLA			
Print Designation	Loading Force F_max_ [N]	Δ F_i_	(Δ F_i_)^2^
PLA_1	2611	20.75	430.56
PLA_2	2601	10.75	115.56
PLA_3	2579	−11.25	126.56
PLA_4	2658	67.75	4590.06
PLA_5	2474	−116.25	13,514.06
PLA_6	2615	24.75	612.56
PLA_7	2508	−82.25	6765.06
PLA_8	2676	85.75	7353.06
Arithmetic mean	2590.25	0	33,507.48
The standard deviation of the maximum loading force F_max_ for the printout			69.19
The resulting value of the maximum loading force F_max_ and its standard deviation			2590.25 ± 69.19 N

**Table 5:** Calculation of the standard deviation of the loading force for the PET-G1 material from the tensile load test.

Material PET-G1			
Print Designation	Loading Force F_max_ [N]	Δ F_i_	(Δ F_i_)^2^
PETG1_1	3906	98.25	9653.06
PETG1_2	3968	160.25	25,680.06
PETG1_3	3917	109.25	11,935.56
PETG1_4	3774	−33.75	1139.06
PETG1_5	3895	87.25	7612.56
PETG1_6	3938	130.25	16,965.06
PETG1_7	3523	−284.75	81,082.56
PETG1_8	3541	−266.75	71,155.56
Arithmetic mean	3807.75	0	225,223.48
The standard deviation of the maximum loading force F_max_ for the printout			179.37
The resulting value of the maximum loading force F_max_ and its standard deviation			3807.75 ± 179.37 N

**Table 6:** Calculation of the standard deviation of the loading force for the PET-G2 material from the tensile load test.

Material PET-G2			
Print Designation	Loading Force F_max_ [N]	Δ F_i_	(Δ F_i_)^2^
PETG2_1	3568	−3.375	11.39
PETG2_2	3513	−58.375	3407.64
PETG2_3	3458	−113.375	12,853.89
PETG2_4	3609	37.625	1415.64
PETG2_5	3638	66.625	4438.89
PETG2_6	3506	−65.375	4273.89
PETG2_7	3579	7.625	58.14
PETG2_8	3700	128.625	16,544.39
Arithmetic mean	3571.375	0	43,003.87
The standard deviation of the maximum loading force F_max_ for the printout			78.38
The resulting value of the maximum loading force F_max_ and its standard deviation			3571.38 ± 78.38 N

**Table 7:** Calculation of the standard deviation of the loading force for the ASA material from the tensile load test.

Material ASA			
Print Designation	Loading Force F_max_ [N]	Δ F_i_	(Δ F_i_)^2^
ASA_1	2986	96.875	9384.77
ASA_2	3110	220.875	48,785.77
ASA_3	2963	73.875	5457.52
ASA_4	2129	−760.125	577,790.02
ASA_5	3103	213.875	45,742.52
ASA_6	3092	202.875	41,158.27
ASA_7	2705	−184.125	33,902.02
ASA_8	3025	135.875	18,462.02
Arithmetic mean	2889.125	0	780,682.91
The standard deviation of the maximum loading force F_max_ for the printout			333.96
The resulting value of the maximum loading force F_max_ and its standard deviation			2889.13 ± 333.96 N

**Table 8:** Calculation of the standard deviation of the loading force for the ABS material from the tensile load test.

Material ABS			
Print Designation	Loading Force F_max_ [N]	Δ F_i_	(Δ F_i_)^2^
ABS_1	2674	−183.75	33,764.06
ABS_2	3020	162.25	26,325.06
ABS_3	3032	174.25	30,363.06
ABS_4	2946	88.25	7788.06
ABS_5	2775	−82.75	6847.56
ABS_6	2791	−66.75	4455.56
ABS_7	2804	−53.75	2889.06
ABS_8	2820	−37.75	1425.06
Arithmetic mean	2857.75	0	113,857.48
The standard deviation of the maximum loading force F_max_ for the printout			127.54
The resulting value of the maximum loading force F_max_ and its standard deviation			2857.75 ± 127.54 N

**Table 9:** Measured values from the deflection test for Original item and PLA and PET-G1 materials.

Original VEX Part		Material PLA		Material PET-G1	
Print Designation	Loading Force F_max_ [N]	Print Designation	Loading Force F_max_ [N]	Print Designation	Loading Force F_max_ [N]
orig_1	146	PLA_1	180	PETG1_1	330
orig_2	144	PLA_2	168	PETG1_2	328
orig_3	125	PLA_3	182	PETG1_3	300
orig_4	134	PLA_4	192	PETG1_4	274
orig_5	130	PLA_5	176	PETG1_5	306
orig_6	136	PLA_6	189	PETG1_6	309
orig_7	126	PLA_7	178	PETG1_7	308
orig_8	116	PLA_8	184	PETG1_8	298
Arithmetic mean	132.125	Arithmetic mean	181.125	Arithmetic mean	306.625

**Table 10:** Measured values from the deflection test for PET-G2, ASA and ABS materials.

Material PET-G2		Material ASA		Material ABS	
Print Designation	Loading Force F_max_ [N]	Print Designation	Loading Force F_max_ [N]	Print Designation	Loading Force F_max_ [N]
PETG2_1	292	ASA_1	212	ABS_1	242
PETG2_2	282	ASA_2	222	ABS_2	240
PETG2_3	290	ASA_3	234	ABS_3	227
PETG2_4	290	ASA_4	226	ABS_4	233
PETG2_5	286	ASA_5	240	ABS_5	223
PETG2_6	283	ASA_6	236	ABS_6	222
PETG2_7	279	ASA_7	221	ABS_7	222
PETG2_8	280	ASA_8	247	ABS_8	212
Arithmetic mean	285.25	Arithmetic mean	229.75	Arithmetic mean	227.625

**Table 11:** Calculation of the standard deviation of the applied force for the original part from the deflection test.

Original VEX Part			
Print Designation	Loading Force F_max_ [N]	Δ F_i_	(Δ F_i_)^2^
orig_1	146	13.875	192.52
orig_2	144	11.875	141.02
orig_3	125	−7.125	50.77
orig_4	134	1.875	3.52
orig_5	130	−2.125	4.52
orig_6	136	3.875	15.02
orig_7	126	−6.125	37.52
orig_8	116	−16.125	260.02
Arithmetic mean	132.125	0	704.91
The standard deviation of the maximum loading force F_max_ for the printout			10.04
The resulting value of the maximum loading force F_max_ and its standard deviation			132.13 ± 10.04 N

**Table 12:** Calculation of the standard deviation of the applied force for the PLA material from the deflection test.

Material PLA			
Print Designation	Loading Force F_max_ [N]	Δ F_i_	(Δ F_i_)^2^
PLA_1	180	−1.125	1.27
PLA_2	168	−13.125	172.27
PLA_3	182	0.875	0.77
PLA_4	192	10.875	118.27
PLA_5	176	−5.125	26.27
PLA_6	189	7.875	62.02
PLA_7	178	−3.125	9.77
PLA_8	184	2.875	8.27
Arithmetic mean	181.125	0	398.91
The standard deviation of the maximum loading force F_max_ for the printout			7.55
The resulting value of the maximum loading force F_max_ and its standard deviation			181.13 ± 7.55 N

**Table 13:** Calculation of the standard deviation of the applied force for the PET-G1 material from the deflection test.

Material PET-G1			
Print Designation	Loading Force F_max_ [N]	Δ F_i_	(Δ F_i_)^2^
PETG1_1	330	23.375	546.39
PETG1_2	328	21.375	456.89
PETG1_3	300	−6.625	43.89
PETG1_4	274	−32.625	1064.39
PETG1_5	306	−0.625	0.39
PETG1_6	309	2.375	5.64
PETG1_7	308	1.375	1.89
PETG1_8	298	−8.625	74.39
Arithmetic mean	306.625	0	2193.87
The standard deviation of the maximum loading force F_max_ for the printout			17.7
The resulting value of the maximum loading force F_max_ and its standard deviation			306.63 ± 17.7 N

**Table 14:** Calculation of the standard deviation of the applied force for the PET-G2 material from the deflection test.

Material PET-G2			
Print Designation	Loading Force F_max_ [N]	Δ F_i_	(Δ F_i_)^2^
PETG2_1	292	6.75	45.56
PETG2_2	282	−3.25	10.56
PETG2_3	290	4.75	22.56
PETG2_4	290	4.75	22.56
PETG2_5	286	0.75	0.56
PETG2_6	283	−2.25	5.06
PETG2_7	279	−6.25	39.06
PETG2_8	280	−5.25	27.56
Arithmetic mean	285.25	0	173.48
The standard deviation of the maximum loading force F_max_ for the printout			4.98
The resulting value of the maximum loading force F_max_ and its standard deviation			285.25 ± 4.98 N

**Table 15:** Calculation of the standard deviation of the applied force for the ASA material from the deflection test.

Material ASA			
Print Designation	Loading Force F_max_ [N]	Δ F_i_	(Δ F_i_)^2^
ASA_1	212	−17.75	315.06
ASA_2	222	−7.75	60.06
ASA_3	234	4.25	18.06
ASA_4	226	−3.75	14.06
ASA_5	240	10.25	105.06
ASA_6	236	6.25	39.06
ASA_7	221	−8.75	76.56
ASA_8	247	17.25	297.56
Arithmetic mean	229.75	0	925.48
The standard deviation of the maximum loading force F_max_ for the printout			11.5
The resulting value of the maximum loading force F_max_ and its standard deviation			229.75 ± 11.5 N

**Table 16:** Calculation of the standard deviation of the applied force for the ABS material from the deflection test.

Material ABS			
Print Designation	Loading Force F_max_ [N]	Δ F_i_	(Δ F_i_)^2^
ABS_1	242	14.375	206.64
ABS_2	240	12.375	153.14
ABS_3	227	−0.625	0.39
ABS_4	233	5.375	28.89
ABS_5	223	−4.625	21.39
ABS_6	222	−5.625	31.64
ABS_7	222	−5.625	31.64
ABS_8	212	−15.625	244.14
Arithmetic mean	227.625	0	717.87
The standard deviation of the maximum loading force F_max_ for the printout			10.13
The resulting value of the maximum loading force F_max_ and its standard deviation			227.63 ± 10.13 N

## Data Availability

The original contributions presented in this study are included in the article. Further inquiries can be directed to the corresponding author.
